# Two *Pellioditis* biocontrol nematode species infect *Ariolimax columbianus*, the Pacific banana slug, and increase mortality in laboratory infectivity trials

**DOI:** 10.1371/journal.pone.0361391

**Published:** 2026-11-11

**Authors:** Emily R. Taylor, Indira Kulkarni Ebner, Dana K. Howe, Casey H. Richart, Rory J. Mc Donnell, Dee Denver

**Affiliations:** 1 Department of Integrative Biology, Oregon State University, Corvallis, Oregon, United States of America; 2 Department of Crop and Soil Science, Oregon State University, Corvallis, Oregon, United States of America; 3 Department of Forest Ecosystems & Society, Oregon State University, Corvallis, Oregon, United States of America; University of Limpopo, SOUTH AFRICA

## Abstract

Gastropods are a highly diverse and often overlooked taxonomic group of ecological and economic importance, with some terrestrial species acting as major agricultural and garden pests. Malacopathogenic nematodes offer an effective biological control method of managing pest slugs and snails as a ‘natural enemy’. *Pellioditis* (syn. *Phasmarhabditis*) *hermaphrodita* and *Pellioditis* (syn. *Phasmarhabditis*) *californica* are two biocontrol nematodes commercialized as Nemaslug® and Nemaslug® 2.0, respectively. Although there is interest in bringing Nemaslug® products to the US, they are currently not permitted due to limited knowledge on their North American distribution and effects on non-target and native species. In this study, we investigated the impact of *P. hermaphrodita* and *P. californica* on *Ariolimax columbianus* across two slug-host life stages, in laboratory infectivity assays. The objectives were to 1. determine whether *P. hermaphrodita* and *P. californica* nematodes impact survival of *A. columbianus*, and 2. evaluate whether there are differential effects on survival in juvenile and adult life stages of *A. columbianus*, in laboratory infectivity trials. We found that *P. hermaphrodita* caused significant mortality in *A. columbianus* with 100% mortality observed in both juvenile and adult slug hosts. The *P. californica* treatment had significant effects on the juvenile *A. columbianus* group only, with 80% mortality, whereas adult mortality was only 8%. In comparison, mortality was 16% in unexposed control juveniles and 4% in control adults. These results indicate that *P. hermaphrodita* and *P. californica* are lethal to the native, non-target Pacific banana slug (*A. columbianus*) under laboratory conditions, with mortality differing between juvenile and adult host life stages. Given the ecological importance of *A. columbianus*, these findings raise concerns for potential non-target effects of *P. californica* and *P. hermaphrodita* on terrestrial gastropod communities and emphasize the need for testing biocontrol agents against multiple host life stages.

## Introduction

Maintaining biodiversity is crucial to an ecosystem’s overall health and stability [1]. Historically, in the world of nonmarine mollusk research, scientific focus has often centered on the eradication of pest species, such as the grey field slug, *Deroceras reticulatum* (O. F. Müller, 1774) (Gastropoda: Agriolimacidae), an invasive estimated to cause $60 million in losses to the state of Oregon’s grass seed industry annually [2]. Consequently, less attention is paid to the many important native species that make up a large part of natural ecosystems [3]. Many species of native terrestrial gastropods (land slugs and snails) play an important role in the ecosystem as detritivores, aiding in nutrient cycling and decomposition which benefits soil health [4]. Insufficient knowledge of the ecology of many native gastropod species hinders efforts to effectively conserve their biodiversity, with mollusks comprising roughly one-third of all recorded animal species extinctions since 1500 CE [5].

In addition to these knowledge gaps, there is a concerning legacy of human-implemented biological control (‘biocontrol’) strategies involving terrestrial gastropods that have resulted in ecological disaster for native species [3,6–8]. A well-known example is the rosy wolfsnail (*Euglandina rosea*) (Férussac, 1821) (Gastropoda: Spiraxidae), a predatory terrestrial snail introduced to Hawaii in the 1950s to control pest species. Rather than controlling target pest populations, *E. rosea* became established in the local ecosystem and has now been widely implicated in the extinction of multiple native Hawaiian snail species, as well as additional extinctions across the Pacific Islands [6–8]. Given this unfortunate history, thorough host range testing of native gastropod species has been identified as a crucial step in assessing effectiveness and safety of biocontrol products [6].

Malacopathogenic nematodes, such as *P. hermaphrodita* (A. Schneider, 1859) (Nematoda: Rhabditidae) and *P. californica* (Tandingan De Ley, Holovachov, McDonnell, Bert, Paine & De Ley, 2016) (Nematoda: Rhabditidae), offer a modern biocontrol solution for eliminating pest slugs from crops and gardens. The nematode *P. hermaphrodita* is a commercialized biocontrol species sold as Nemaslug® in Canada, Europe, and Kenya [9], with the purpose of controlling target pests such as *D. reticulatum*. The nematode *P. californica* is another species that has shown increasing potential as a replacement for *P. hermaphrodita* and is currently sold as the new and improved Nemaslug® 2.0 [9,11]*.*

While not currently permitted for sale in the US, there is increasing interest in bringing Nemaslug® to the US market as a natural alternative to current chemical control methods such as metaldehyde pellets, which can be ineffective and potentially harmful to the environment [10,12]. The lethality of *P. hermaphrodita* to pest *D. reticulatum* is well documented [9], and several potential non-target gastropod species have been evaluated in Europe [13]. However, considerably less research has examined gastropods native to the United States.

Denver et al. [14] recently demonstrated that *P. hermaphrodita* infects and increases mortality in the Pacific sideband snail, *Monadenia fidelis* (J. E. Gray, 1834) (Gastropoda: Monadeniidae*)*. The study tested multiple strains and exposure rates of *P. hermaphrodita* and found that individuals with larger shell diameters had higher survival than smaller individuals [14]. However, susceptibility to malacopathogenic nematodes can vary among host taxa, and results from one native gastropod species cannot necessarily be extended to another. Denver et al. [14] evaluated a snail host and only *P. hermaphrodita*; therefore, the susceptibility of native slug species and their responses to *P. californica* remain important areas for further investigation.

Previous infectivity studies, primarily focused on species of economic relevance, have found *P. hermaphrodita* to be lethal to a diverse range of terrestrial gastropods, including slugs belonging to the families Arionidae, Limacidae, and Milacidae, and Testacellidae [9,11,16–19], as well as several snail species [9,13,19–21]. The nematode *P. californica* has been found to cause mortality in the slug *D. reticulatum* and the White garden snail (*Theba pisana*) (O. F. Müller, 1774) (Gastropoda: Helicidae), the amber snails (*Succinea* spp.), and neonate brown garden snails (*Cornu aspersum*) (O. F. Müller, 1774) (Gastropoda: Helicidae) [11,20–23]. Susceptibility to *Pellioditis* infection can also vary with host size or life stage. For example, juvenile *Arion lusitanicus* Mabille, 1868 (Gastropoda: Arionidae) have been reported to be more susceptible to *P. hermaphrodita* infection than adults [17,24]. To date, no slugs within family Ariolimacidae have been tested.

The non-target focus of this study is the Pacific banana slug (*Ariolimax columbianus*) (A. A. Gould, 1851) (Gastropoda: Ariolimacidae) (Fig 1). *A. columbianus* is a North American Pacific coast native gastropod that can be found ranging from Alaska to northwestern California. They are the largest gastropod species found on the North American continent [15]. In addition to their vital role in the ecosystem, banana slugs are recognized as familiar cultural icons of the North American West coast. Their status as a native, non-target, and well-known species makes them a suitable candidate for non-target testing in this study.

**Fig 1:**
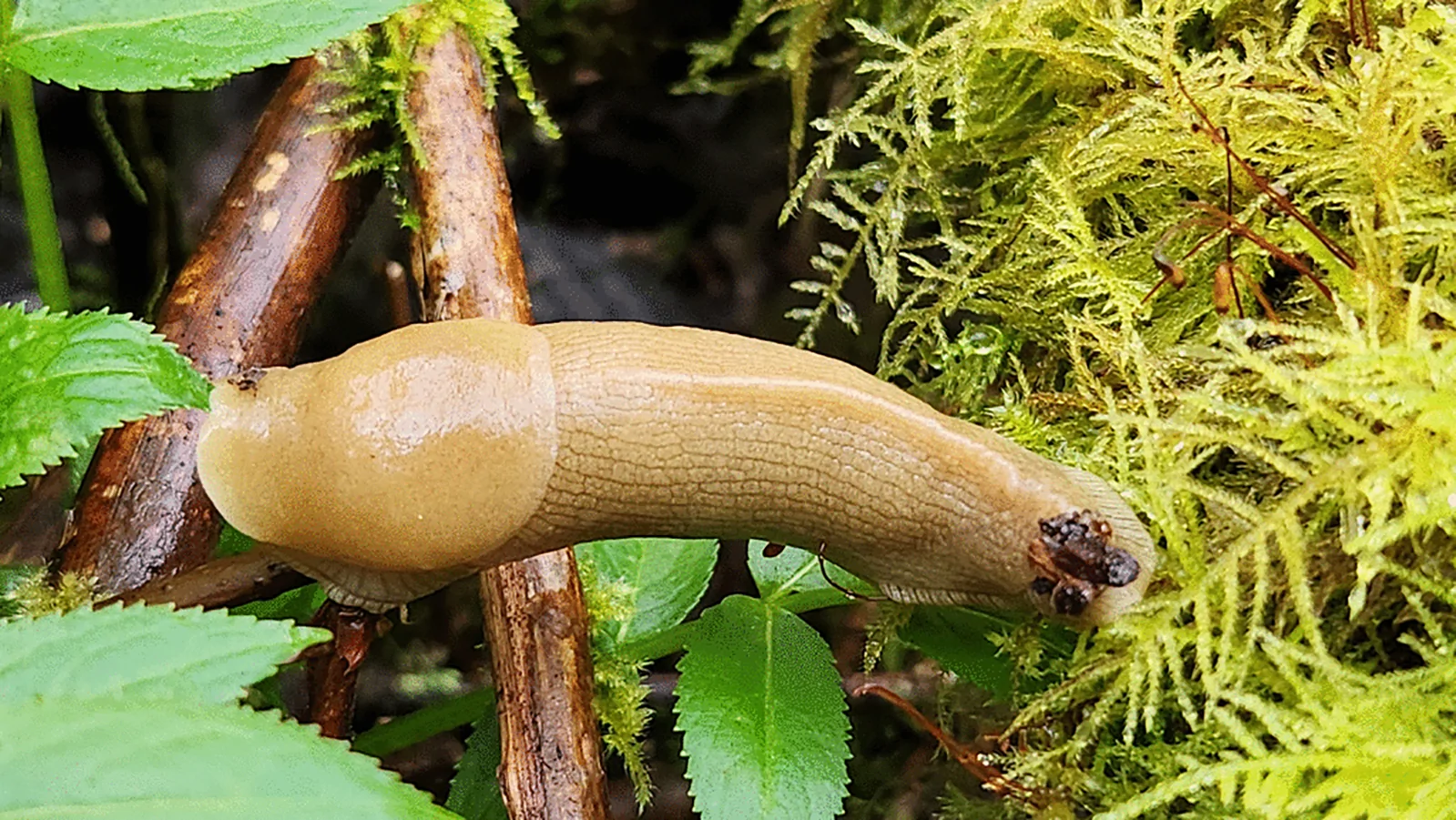
*A. columbianus* in its natural ecology on the Oregon coast. Photo by C.H. Richart.

In this study, we investigated the effects of *P. hermaphrodita* and *P. californica* on the survival of juvenile and adult *A. columbianus* in controlled laboratory infectivity trials. We tested whether exposure to either nematode species increased mortality relative to untreated controls and whether susceptibility differed between host life stages. By examining a previously untested host family, directly comparing two malacopathogenic nematode species, and evaluating defined juvenile and adult life stages, this study addresses a distinct gap in the assessment of biocontrol nematode host range and potential non-target effects in Pacific Northwest ecosystems.

## Materials and methods

### Slugs and nematodes

*Ariolimax columbianus* samples were collected along the Oregon coast from March 2024 to April 2024. As per Oregon Department of Agriculture policies, permits were not required to collect *A. columbianus* (a non-threatened terrestrial gastropod species) from public lands. Individuals were identified as juveniles or adults based on weight, with juveniles ranging from 0.22-2.67g, and adults ranging from 6.60-30.7g (Fig 2). These ranges were comparable to weight group classifications described in Pearson et al. [25]. Weight range outliers were not included in this study. All specimens were kept in plastic containers and fed organic sweet potatoes, as outlined in Mc Donnell et al. [11], before and during infectivity trials. Slug specimens were brought into the lab and kept in an incubator at 18℃ with a 12-hour photoperiod until trials began.

**Fig 2:**
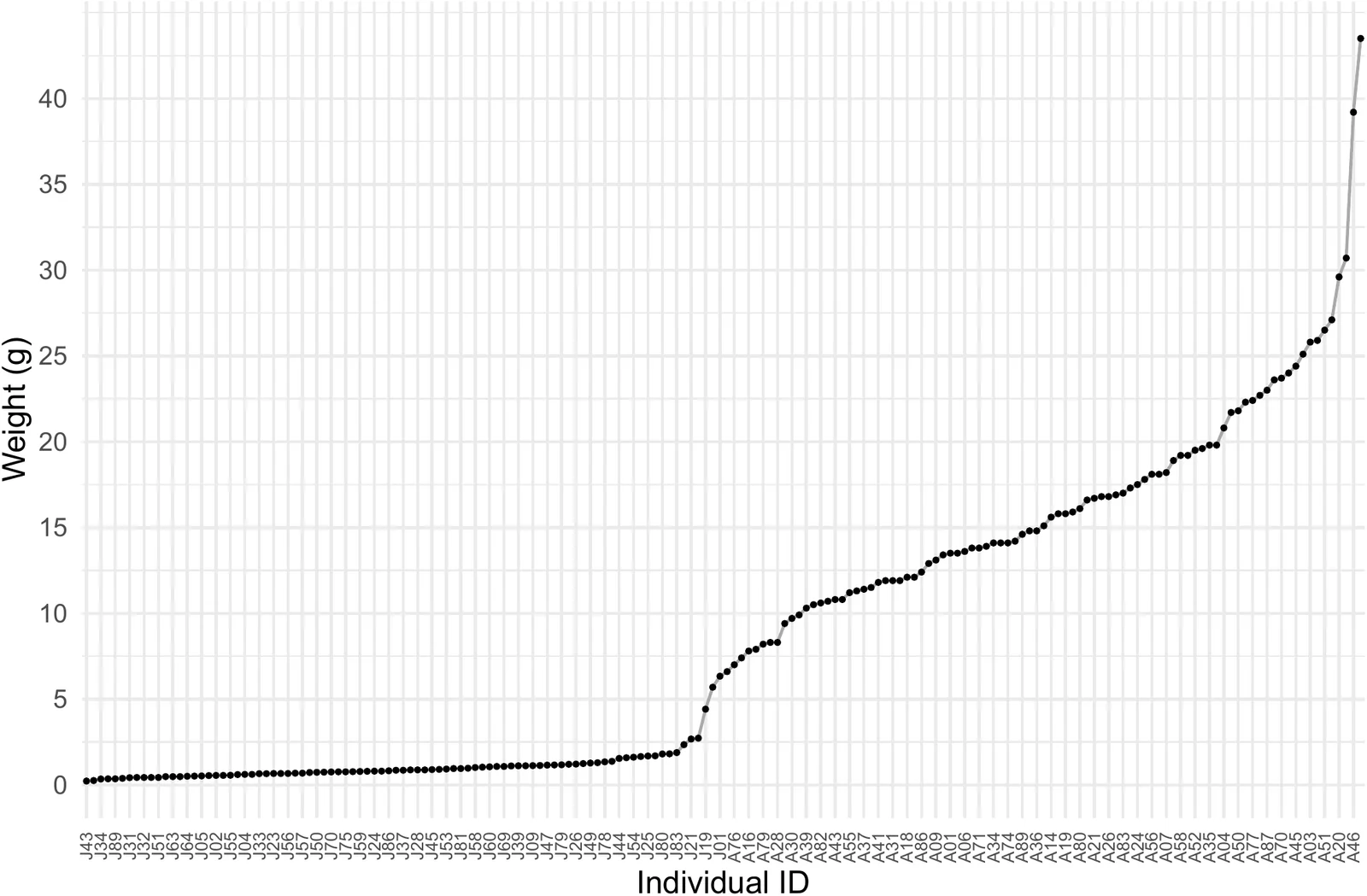
*Ariolimax columbianus* rank-ordered weight distribution. Weight in grams (y-axis) of each *A. columbianus* individual (x-axis) collected for the trial (n = 178), arranged in ascending order to illustrate size variation. Individual IDs (x-axis) indicate juvenile or adult classification followed by a unique numerical identifier (‘J’ for juveniles; ‘A’ for adults).

The Oregon-derived *P. hermaphrodita* strain DL309 and the Washington-derived *P. californica* strain DL320 were used in this study. We also note here that while *P. hermaphrodita* and *P. californica* were previously classified in the *Phasmarhabditis* genus, recent work has led to its dissolution, with ‘*Pellioditis*’ now replacing ‘*Phasmarhabditis’* as the genus for this group of nematode species [26]. These nematodes were grown, collected, and washed using the modified White traps protocol to select for infective juvenile stage nematodes [27]. Infective juvenile stage nematodes (IJs) of each strain were aliquoted into individual 5mL suspensions at the BASF recommended application rate of 30 IJs/𝑐𝑚2.

### Infectivity assays

Infectivity assays took place in plastic containers with a bottom layer of autoclaved soil, in two different container sizes corresponding to life stage. Juvenile *A. columbianus* were housed in round plastic containers 64 ${cm}^{\text{2}}$ in area with 25g of autoclaved soil, as described in Denver et al. [14]. Adult *A. columbianus* were housed in rectangular plastic containers 435 ${cm}^{\text{2}}$ in area with 170g of autoclaved soil. This trial included three experimental groups: *P. hermaphrodita* (strain DL309) treatment, *P. californica* (strain DL320) treatment, and negative control treatment where no nematodes were introduced. Each treatment was applied to one group of adult slugs and one group of juvenile slugs. Nematodes were suspended in 5 mLs of deionized (DI) water and applied evenly across the soil surface in each treatment container at the BASF recommended application rate of 30 IJs/𝑐𝑚2. Control group containers received 5mL of DI water without nematodes. Five slugs of the same life stage were added to each container, and a total of five replicates (five containers) per treatment group were used, for a total of 30 assay arenas. Slugs were fed organic sweet potato every two days, with uneaten food removed after 24 hours.

Infectivity trial containers were maintained in a Thermo Precision Model 818 growth chamber at 18℃ with a 12-hour photoperiod. During the infectivity trial, containers were checked daily and monitored for mortality until day 38 of the experiment, in which the trial was ended. Mortality was assessed following a modified version of the procedure outlined in McDonnell et al. [11], including assessment of physical cues such as discoloration, body position (e.g., laying on their side), and lack of behavioral response to eyestalks stimulated with a metal spatula.

### Statistical analyses

Slug survival data was initially visualized by plotting total percent mortality over time (38 days total) for each treatment group. To formally assess survivorship differences, Kaplan-Meier survival curves were generated using the survival package in RStudio (version 2025.05.1 + 513) for each treatment group. This non-parametric method of analysis allowed for right-censored observations, i.e., slugs that remained alive by the end of the trial, to be accounted for. Pairwise differences in survivorship between treatment groups were evaluated using log-rank tests, with an alpha level of 0.05 and p-values adjusted for multiple comparisons using the Bonferroni correction (see Table 1).

**Table 1:** Adjusted pairwise log-rank test p-values for survival comparisons between treatment groups.

Treatment	Adult Ctrl	Adult Ph	Adult Pc	Juv Ctrl	Juv Ph	Juv Pc
Adult Ctrl						
Adult Ph	2.7e-11*					
Adult Pc	1.0	6.6e-12*				
Juv Ctrl	1.0	2.7e-11	1.0			
Juv Ph	2.6e-11	5.8e-13*	5.8e-13	8.2e-13*		
Juv Pc	5.4e-07	1.0	6.8e-07*	4.6e-06*	0.16	

Tests were adjusted for multiple comparisons using the Bonferroni correction. Significant differences (p < 0.05) are indicated with an asterisk.

## Results

The results of the laboratory infectivity assay demonstrated that both *Pellioditis* nematode species significantly increased mortality in at least one life stage of *A. columbianus*, as compared to negative controls (Table 1). During the trial, nematodes could be seen actively infecting *A. columbianus* specimens, observed through a dissecting microscope on the outer surface of the mantle and eventually in large numbers on the slug cadaver (Fig 3). *P. hermaphrodita* was found to have significant effects on survival in both life stage groups (juvenile and adult), while *P. californica* only significantly impacted survival in the juvenile group. Overall, *P. hermaphrodita* killed host slugs at a faster rate than *P. californica* (Fig 4). The first slug mortalities occurred on Day 5, with one slug death each in the juvenile *P. hermaphrodita* group and the juvenile *P. californica* group (Fig 4). The juvenile *P. hermaphrodita* treatment was the first to experience complete mortality in 16 days. The adult *P. hermaphrodita* group also reached complete mortality, at 27 days. Although death occurred in both, neither the juvenile nor adult *P. californica* treatment groups reached complete mortality by the conclusion of the trial.

**Fig 3:**
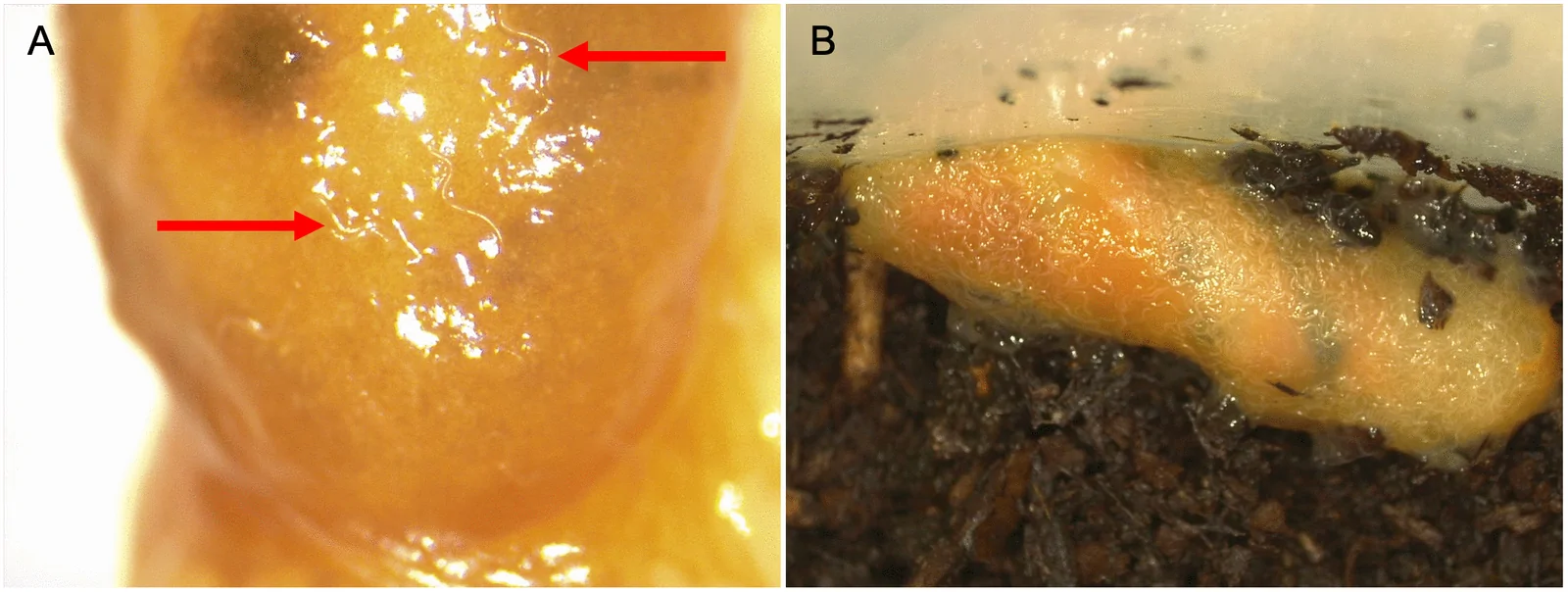
*P. hermaphrodita*-infested juvenile *A. columbianus* individual from the laboratory infectivity trial. **(A).** Several *P. hermaphrodita* nematodes are visible on the outer surface of a juvenile *A. columbianus* mantle under a dissecting microscope, as indicated by red arrows. **(B).** Thousands of *P. hermaphrodita* nematodes seen on the cadaver of a juvenile *A. columbianus*.

**Fig 4:**
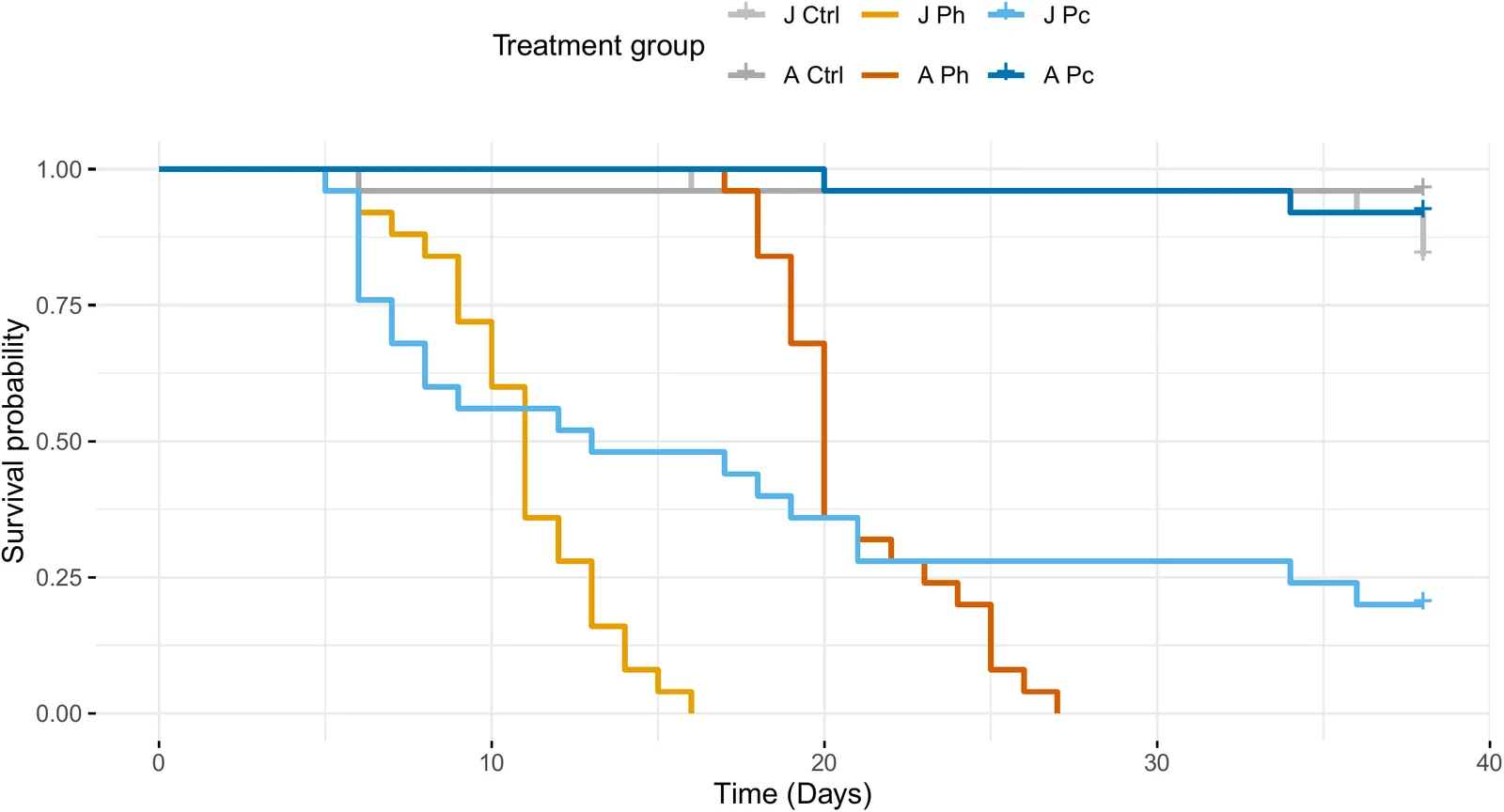
Kaplan-Meier survival probability (y-axis) as a function of number of exposure days (x-axis). Results for each treatment group are shown (Juvenile control, light grey; Adult control, dark grey; Juvenile *P. hermaphrodita*, light orange; Adult *P. hermaphrodita*, dark orange; Juvenile *P. californica*, light blue; Adult *P. californica*, dark blue).

## Discussion

Both *P. hermaphrodita* and *P. californica* increased mortality in the Pacific coast-native, non-target slug *A. columbianus* under laboratory conditions (Fig 4). The strength of this effect differed between nematode species and host life stages (Table 1). *P. hermaphrodita* caused complete mortality in both juvenile and adult slugs, whereas *P. californica* caused substantial mortality in juveniles but comparatively little mortality in adults. These results demonstrate that susceptibility of *A. columbianus* to *Pellioditis* nematodes is dependent on both the nematode species and the life stage of the host.

The stronger effect of *P. hermaphrodita* relative to *P. californica* is consistent with previous studies comparing these nematodes. In *D. reticulatum, P. hermaphrodita* has been reported to cause mortality more rapidly than *P. californica* [11]. The contrast between nematode species was most prominent in the adult *A. columbianus*: *P. hermaphrodita* caused complete mortality, whereas *P. californica* exposure resulted in high survival comparable to that of the control group. This suggests that the two nematode species may differ in their ability to cause mortality in larger or later-stage *A. columbianus*. Differences in virulence, host compatibility, or other aspects of the nematode-host system could contribute to this result, although the underlying mechanisms of these differences were not evaluated in the present study.

Host life stage was also associated with survival. Juvenile *A. columbianus* experienced mortality more rapidly than adults in both nematode treatments (Fig 4), a difference that is especially prominent in the *P. californica* treatment. Size- or life stage-dependent susceptibility has been observed in other terrestrial gastropods exposed to *Pellioditis* nematodes, including *D. reticulatum* and *A. lusitanicus* [17,24], with smaller or younger individuals generally exhibiting greater susceptibility than larger individuals. These studies indicate that host size or developmental stage can influence susceptibility, although this varies among host and nematode species. Testing only one life stage may therefore underestimate potential effects on a non-target population.

The susceptibility of adult *A. columbianus* to *P. hermaphrodita* is especially notable given the large body size of this species. Adult banana slugs in this study included individuals substantially larger than many gastropod hosts evaluated in previous *Pellioditis* studies, with some individuals approaching approximately 30g (Fig 2). Despite this, adult *A. columbianus* exposed to *P. hermaphrodita* experienced complete mortality. This suggests that size alone may not provide protection against *Pellioditis* infection, and may be dependent on other host- and nematode-specific factors.

These findings also expand on previous non-target infectivity testing in the native snail *M. fidelis*, in which *P. hermaphrodita* increased mortality under laboratory conditions [14]. Together, the results demonstrate that susceptibility to malacopathogenic nematodes is not limited to economically important pest species and can occur in native North American taxa. However, this result should not be generalized across gastropod species. The present study tested a slug rather than a snail and found differences associated with both nematode species and host life stage. These differences reinforce the value of host-range testing across diverse non-target species.

Previous assessments of *P. hermaphrodita* in Europe have generally concluded that it poses limited risk to tested non-target gastropods [9,13], while considerably less information is available regarding potential non-target effects of *P. californica* or either species within North American gastropod communities. The gastropod species most likely to encounter commercial applications in North America may also likely differ from those evaluated in European agricultural and hedgerow environments. Therefore, additional testing of native species likely to overlap with nematode application areas would improve our understanding of potential non-target effects.

The present experiment was conducted under controlled laboratory conditions and therefore establishes susceptibility rather than true ecological risk of *Pellioditis* exposure in the field setting. Environmental contexts, host behavior, nematode survival, and routes of exposure may have a large effect on outcomes. Mesocosm and field-based experiments would help determine whether the mortality observed here occurs under more environmentally realistic conditions. Future studies could also evaluate sublethal responses, including changes in growth, behavior, reproduction, or immune function, which have not been explored by the mortality-based assay used here.

In conclusion, *A. columbianus* was susceptible to both *P. hermaphrodita* and *P. californica* under laboratory conditions, with responses differing substantially between nematode species and host life stages. The complete mortality of adult banana slugs exposed to *P. hermaphrodita*, despite their large body size, further suggests that susceptibility cannot be predicted from body size alone. Our findings advocate for broader host-range assessment of malacopathogenic nematodes across multiple native gastropod taxa and life stages for accurate and thorough understanding of potential ecological effects accompanying biocontrol nematode application.
